# Determinants of COVID-19 Vaccine Acceptance among the Adult Population of Bangladesh Using the Health Belief Model and the Theory of Planned Behavior Model

**DOI:** 10.3390/vaccines9121393

**Published:** 2021-11-25

**Authors:** Muhammad Mainuddin Patwary, Mondira Bardhan, Asma Safia Disha, Mehedi Hasan, Md. Zahidul Haque, Rabeya Sultana, Md. Riad Hossain, Matthew H. E. M. Browning, Md. Ashraful Alam, Malik Sallam

**Affiliations:** 1 Environment and Sustainability Research Initiative, Khulna 9208, Bangladesh; raju.es111012@gmail.com (M.M.P.); mondirabardhan.22@gmail.com (M.B.); dishaa@vscht.cz (A.S.D.); rtr.zahid@gmail.com (M.Z.H.); 2 Environmental Science Discipline, Life Science School, Khulna University, Khulna 9208, Bangladesh; rabeya.sultana@ku.ac.bd; 3 Environmental Technology and Engineering, University of Chemistry and Technology, 16628 Prague, Czech Republic; 4 Department of Environmental Science and Disaster Management, Bangabandhu Sheikh Mujibur Rahman Science and Technology University, Gopalganj 8100, Bangladesh; mehedi.esd16@bsmrstu.edu.bd; 5 Institute of Disaster Management, Khulna University of Engineering & Technology, Khulna 9203, Bangladesh; riad.hossain@idm.kuet.ac.bd; 6 Department of Parks, Recreation and Tourism Management, Clemson University, Clemson, SC 29634, USA; mhb2@clemson.edu; 7 Department of Global Health Policy, Graduate School of Medicine, The University of Tokyo, Tokyo 113-0033, Japan; aalam@m.u-tokyo.ac.jp; 8 Department of Pathology, Microbiology and Forensic Medicine, School of Medicine, The University of Jordan, Amman 11942, Jordan; 9 Department of Clinical Laboratories and Forensic Medicine, Jordan University Hospital, Amman 11942, Jordan

**Keywords:** coronavirus, vaccine acceptance, Health Belief Model, Theory of Planned Behavior, Bangladesh

## Abstract

Vaccination is undoubtedly one of the most effective strategies to halt the COVID-19 pandemic. The current study aimed to investigate the acceptance of COVID-19 vaccination and its associated factors using two health behavior change frameworks: the Health Belief Model (HBM) and the Theory of Planned Behavior (TPB). A total of 639 Bangladeshi adults (mean age: 24 years) participated in a cross-sectional online study between July and August 2021. The questionnaire covered questions regarding vaccine intentions, sociodemographic features, health status, perceived trust in/satisfaction with health authorities, reasons for vaccine hesitancy, and factors related to the health behavior change frameworks. Hierarchical logistic regression was employed to determine associations between these predictors and vaccine acceptance. The intention to get a COVID-19 vaccination was expressed among 85% of the participants. In fully adjusted models, students and respondents with more normal body weights reported higher intentions to get vaccinated. Respondents were also more likely to seek vaccination if they reported greater levels of perceived susceptibility, benefits, and cues to action, as well as lower levels of barriers and self-efficacy. Fear of future vaccine side effects was the most common reason for COVID-19 vaccine hesitancy and was expressed by 94% of the vaccine-hesitant respondents. These factors should be considered by health authorities in Bangladesh and perhaps other countries when addressing the plateauing COVID-19 vaccination rates in many populations.

## 1. Introduction

The coronavirus disease 2019 (COVID-19) has been designated as a pandemic and a public health emergency of international concern (PHEIC) [1]. The pandemic has resulted in more than 4.9 million deaths across 220 countries as of 28 October 2021 [2]. Bangladesh announced its first clinically verified COVID-19 case on 8 March 2020 [3]. At the time of writing, Bangladesh has recorded 1,568,857 COVID-19 cases and 27,847 deaths, which have placed a great burden on human mental and physical health, as well as on economic sustainability in the country [2].

To cope with this situation, countries around the globe have adopted stringent measures and restrictions to limit the spread of the virus, which include but are not limited to social isolation, obligatory use of face masks, and travel restrictions [4]. Such stop-and-go strategies are expected to continue until herd immunity has been achieved. However, the most promising strategy to control the pandemic is a vaccine with an established track record of efficacy and a successful vaccination rollout [5].

Soon after the severe acute respiratory syndrome coronavirus 2 (SARS-CoV-2) was identified, many research institutions and pharmaceutical companies began developing vaccines and conducting clinical trials to evaluate their safety and efficacy [6]. In December 2020, dozens of COVID-19 vaccines gained approval for commercialization in multiple countries. These included such brand names as Pfizer–BioNTech, Moderna, Janssen, Sinopharm-BBIBP, Sputnik V, CoviVac, and Covaxin [7]. The Bangladeshi government ordered and paid for approximately 30 million doses of the Oxford–AstraZeneca vaccine, and these have been delivered in installments throughout 2021. Bangladesh is also slated to receive 68 million doses of the Covax vaccine through the World Health Organization (WHO) and Gavi, the Vaccine Alliance [8]. Despite the considerable progress of these vaccination rollouts, vaccine hesitancy remains a major issue and stands as a barrier to mass vaccination success [9,10].

Most of the earlier studies on vaccine hesitancy come from developed countries, including the USA, Canada, Saudi Arabia, and China. A global study conducted in 19 countries in June 2020 found that 71.5% of participants were likely to get vaccinated against COVID-19 [11]. A recent study conducted among older adults from southern Italy reported that 92.7% participants were willing to be vaccinated, with a similarly high acceptance rate of 91.9% reported among Italian undergraduate students [12,13]. Comparable findings were observed in other countries, including Canada, Saudi Arabia, the United States, and China, where acceptancy rates ranged from 67% to 91% [14,15,16,17,18]. Much less is known about vaccine hesitancy in low/middle-income countries [9].

However, some initial research has estimated acceptance rates of the COVID-19 vaccine in Bangladesh. A survey that was conducted with approximately 1650 adult participants found that 57% showed COVID-19 vaccine hesitancy [19]. Another survey with 3646 adults found that 75% expressed willingness to accept a COVID-19 vaccination, when it was shown to be safe and effective and when it was provided for free [20]. Vaccine hesitancy in Bangladesh has been found to be more common among the elderly, farmers, day laborers, homemakers, and less well-educated people in rural, semi-urban, and slum areas [20]. One study has also explored the behavioral determinants of COVID-19 vaccine acceptance amongst Dhaka City residents [21].

Identifying vaccine hesitancy rates and factors associated with vaccine hesitancy is critical for successful vaccination rollouts, especially in developing countries that are continuously struggling with emerging infectious diseases [22]. Recently in Bangladesh, a survey found that 46% of respondents believed COVID-19 vaccines had adverse effects and 16% felt they had life-threatening side effects [19]. Personal views, lack of trust, religious reasons, conspiracy beliefs, and safety concerns can contribute to widespread misconceptions about vaccines [14,19,23,24]. However, the determinants of vaccine acceptance and hesitancy vary across time, place, and specific vaccines. As a result, the precise roots of hesitancy with respect to vaccines are poorly understood [25].

Public trust and satisfaction with government health authorities are factors that may be used to help understand vaccine hesitancy [26,27]. Adopting various preventive measures during the early phase of the COVID-19 pandemic, such as social distancing and non-pharmaceutical interventions, have been associated with trust in governments [28,29]. These factors were eroded for some individuals when governmental health authorities were criticized for improperly supporting healthcare systems and responding slowly to the pandemic [30].

The Health Belief Model (HBM) and the Theory of Planned Behavior (TPB) are additional tools to explain vaccine hesitancy [31,32,33]. In the context of the COVID-19 vaccination, the tenants of the HBM suggest that individuals with greater perceived threats of getting infected in the absence of a vaccine (“perceived susceptibility”), beliefs that an infection would present a serious health concern (“perceived severity”), perceptions that the vaccine would be effective in preventing a serious infection (“perceived benefits”), beliefs that the vaccine would not cause adverse effects (“perceived barriers”), perceptions that health authorities recommend the vaccination (“cues to action”), and those who participate in other health behaviors, such as exercise and a healthy diet (“health motivation”), would be more likely to intend to get vaccinated. The tenants of the TPB extend these predictors of vaccine acceptance to include positive attitudes towards the vaccine (“attitudes”), presence of individuals important to the respondent who support/encourage vaccination (“subjective norms”), perceived behavioral control entailing the perceived easiness/difficulty to perform a behavior, and self-efficacy. Thus, these two models can collaboratively predict behavioral intentions based on attitudes and perceptions [34,35].

Bangladesh has experienced a high transmission rate of COVID-19 and now faces a deadly second wave of the pandemic due to the highly contagious Delta variant [36]. To our knowledge, no research in Bangladesh has employed the HBM and TPB models to explain vaccine hesitancy despite their strong psychometric properties and broad relevance to explaining health behavior. Thus, the aim of the current study was to determine the potential factors of COVID-19 vaccine hesitancy/acceptance among Bangladeshi adults using the HBM and TPB models.

## 2. Materials and Methods

### 2.1. Study Settings and Participants

We employed a cross-sectional online survey that was administered between 5 July and 5 August 2021, in Bangladesh. This time period corresponded with a devastating second wave of infections across the country, before mass vaccination was available. The study population was Bangladeshi residents aged 18 or over living in Bangladesh at the time of the survey and without their first dose of a COVID-19 vaccination. The data were collected with a convenience sampling method. To minimize selection bias, we recruited a diversified set of participants and collected data from all eight administrative divisions of Bangladesh. The survey was constructed using Google Forms, and the survey link was distributed through attainable social networks, including Facebook, WhatsApp, LinkedIn, and Instagram. Participants were informed that their response was voluntary and were requested to share the Google link with their acquaintances.

A group of relevant experts were consulted to assess the relevancy of the questionnaire. The content validity of a questionnaire was measured with a relevancy assessment. Experts provided careful feedback on the intelligibility, relativeness, and implications of the questionnaire to achieve the objectives of the study. We then performed a pilot study with 40 participants to receive additional feedback on the questionnaire. Respondents were asked to rate each question on a 7-point scale ranging from 1 (not meaningful) to 7 (very meaningful). Participants’ feedback was used to modify the original questionnaire and make it more comprehensible. The reliability index of the pilot and final questionnaire was determined using Cronbach’s alpha values. All values were over 0.78, indicating satisfactory levels of reliability.

Previous studies using our suite of measures were unavailable; therefore, we used an online calculator to estimate our necessary sample size [37]. An online sample size calculator (https://statulator.com/ accessed on 17 November 2021) determined we needed at least 427 respondents based on a 10% non-response rate, 5% precision, and 50% proportion, with a 95% confidence range for the overall population size of 164.7 million people.

The survey response rate was 100% for each item. Hence, missing data analysis was not required. A total of 639 responses were collected. However, 96 participants were not eligible for participation since they had already been vaccinated against COVID-19. Thus, a total of 543 respondents comprised the study sample that was used for the final analyses.

Electronic consent was obtained from all participants prior to their completion of the survey. Participants could opt out of submitting the completed form. The survey did not ask participants to provide their names or email addresses, ensuring that participants could not be identified. The research ethical clearance board of the Institute of Disaster Management, Khulna University of Engineering & Technology, Khulna, Bangladesh waived the approval for this study.

### 2.2. Measures

**Figure 1:**
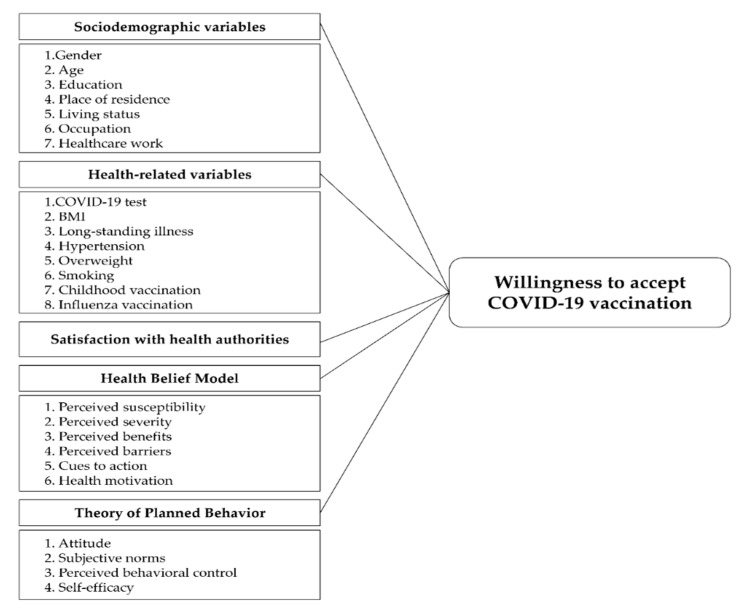
Conceptual framework of factors predicting COVID-19 vaccination intentions. COVID-19: Coronavirus disease 2019; BMI: Body mass index.

A structured questionnaire was formulated and used to collect data from each respondent. The questionnaire asked about their sociodemographic characteristics, health-related characteristics, intentions to receive a COVID-19 vaccine, health belief measures, satisfaction with health authorities, and reasons for refusing a COVID-19 vaccination. Health belief measures were adapted from the HBM and TPB [38,39]. Overall, 49 questions were asked to respondents. A conceptual model describing the parameters comprising the study measurements is shown in Figure 1.

#### 2.2.1. Willingness to Accept Vaccine and Reasons Not to Accept Vaccine

Participant’s willingness to accept a COVID-19 vaccine was measured with a single item. Respondents were asked “Will you take the COVID-19 vaccine when it becomes available?” with ‘Yes’, ‘No’ and ‘Not sure’ response options. Participants were categorized into three groups: (1) intended to take the vaccine (response = ‘Yes’), (2) not sure about taking the vaccine (response = ‘Not sure’), and (3) reluctant to take the vaccine (response = ‘No’). We considered ‘No’ and ‘Not sure’ responses as expressions of vaccine hesitancy. For these respondents, we asked why they would refuse/hesitate to take the vaccine. Options included “I believe that COVID-19 is not dangerous to me”, “Natural immunity lasts longer than a vaccination”, “I prefer other people get the vaccine first”, “I doubt the vaccine’s effectiveness”, “I fear the unknown effects of vaccines in the future”, “I have contraindications to the vaccine (e.g., allergies, high blood pressure, diabetes”, “I have insufficient information regarding the vaccine”, and “the financial cost is a hindrance if the vaccine is not free”. Respondents were asked to indicate one or more of these potential reasons.

#### 2.2.2. Trust/Satisfaction with Health Authorities

Public trust and satisfaction with the country’s health authorities to control COVID-19 transmission was assessed with two items (α = 0.72). These were “I am satisfied with the country’s health authorities to control the spread of COVID-19” and “I am satisfied with the country’s political leadership on COVID-19 control”. Respondents were asked to rate each item on a 5-point scale from strongly disagree (1) to strongly agree (5). An overall score was calculated by averaging the two scores, with higher scores indicating greater trust/satisfaction with the health authorities of the country.

#### 2.2.3. Health Belief Model (HBM) Variables

The HBM is composed of six dimensions, including perceived susceptibility, perceived severity, perceived benefits, perceived barriers, cues to action, and health motivation. Perceived susceptibility was measured with two items (α = 0.92), including “I may get infected if I do not get a COVID-19 vaccination” and “My family may get infected if they do not get a COVID-19 vaccination”. Perceived severity included “Complications from COVID-19 are very serious” and “Recovering from COVID-19 would take a long time” (α = 0.61). Perceived benefits included two items: “The vaccine will reduce my fear of infection” and “The vaccine will be highly effective to prevent the spread of COVID-19” (α = 0.79). Perceived barriers were also assessed with two items: “The COVID-19 vaccine would have possible side effects" and “I am doubtful about the efficacy of the COVID-19 vaccine” (α = 0.76). The second item was based on previous research that applied the HBM to understanding COVID-19 vaccine hesitancy; this research identified unique contributions of efficacy concerns as a barrier to vaccine acceptance and beliefs in efficacy as a perceived benefit of vaccine acceptance [34]. Cues to action included four items: “I would take the vaccine if recommended by a doctor”, “I would take the vaccine if recommended by a public figure/political leader”, “I would take the vaccine if the Ministry of Health publishes any guideline”, and “I would take the vaccine if recommended by family/friends” (α = 0.80). Health motivation was measured with two items: “I exercise regularly as recommended by my age range” and “I make sure to eat a healthy and varied diet” (α = 0.72).

Respondents were asked to rate all HBM items on a five-point scale ranging from 1 (strongly disagree) to 5 (strongly agree). An aggregate score for each dimension was obtained by averaging the item scores; higher scores indicate greater levels of a specific dimension. An exception to this rule was the perceived barrier scale, which was reverse coded; higher perceived barrier scores indicated lower levels of perceived barriers.

#### 2.2.4. Theory of Planned Behavior (TPB) Variables

The original Theory of Planned Behavior (TPB) was conceptualized in three dimensions, but more recent research on health behavior change has conceptualized it with four dimensions: attitudes, subjective norms, perceived behavior control (PBC), and self-efficacy. Attitudes were measured using a single item according to past research [32]: “Getting vaccinated is a tedious and time-consuming process”. Subjective norms were assessed with two items: “Most of my friends will support the COVID-19 vaccine” and “My family and friends will respond positively to vaccination” (α = 0.89). The last two variables were measured with one item each according to past research [32,40], and included the “The decision to get vaccinated is entirely up to me” for PBC and “If I take all the necessary precautions (disinfection of hands, etc.) I do not need to be vaccinated” for self-efficacy. The self-efficacy item was reverse coded. All items were evaluated on a 5-point response scale ranging from 1 (strongly disagree) to 5 (strongly disagree). Item responses were averaged to obtain aggregated scores for each specific dimension of TPB.

#### 2.2.5. Sociodemographic and Health Variables

Sociodemographic variables included gender, age, education, residency, living status, occupation, and healthcare worker status. Respondents were asked about their educational level using three bins: (1) currently enrolled in college or having less than a college education, (2) completed a bachelor’s degree, or (3) completed higher levels of education beyond a bachelor’s degree. Place of residence was classified by respondents as currently living in an urban or in a rural area. Living status was assessed by asking whether participants lived with family members, non-family members, or alone. Occupation was classified by the respondent as currently unemployed, student or working in the public sector, working in the private sector, or self-employed. Healthcare worker status was classified into three groups: a healthcare professional, a healthcare student, or neither a healthcare professional nor student.

Health-related variables included COVID-19 test positivity, body mass index (BMI), diagnosis of being overweight, having a long-standing illness(es), hypertension status, smoking habits, childhood vaccinations, and seasonal influenza vaccinations. COVID-19 susceptibility, long-standing illness, overweight status, hypertension status, smoking habit and childhood vaccination status were assessed by asking respondents to indicate ‘Yes’ or ‘No’. Body Mass Index (BMI) was calculated with the respondent’s height (m^2^) and weight (kg). BMI was then classified into four categories: underweight (<18.5), normal weight (18.5–24.9), overweight (25.0–29.9), and obesity (>30.0). Respondent’s seasonal influenza vaccination status was evaluated by asking them whether they had never taken it, taken it in the last year, taken it in the current flu season, taken it annually, or could not remember.

### 2.3. Statitical Analysis

Participants were divided into three groups based on their vaccination intentions: those who would receive the vaccine, those who were uncertain, and those who were reluctant to receive the vaccine. For subsequent analysis, the last two categories were combined into a single group: “undecided/unwilling”. When performing statistical analysis on vaccination intentions, we used two groups rather than three to emphasize the potential distinction between those who intended to accept a COVID-19 vaccine and those who did not, as well as to identify factors that predicted one’s intention to vaccinate. Chi-square tests for categorical variables and Kruskal–Wallis tests for continuous variables were used to identify statistically significant differences between the vaccine acceptance group and the unwillingness/undecided group based on sociodemographics, health-related factors, trust and satisfaction, and HBM and TPB dimensions.

A hierarchical logistic regression analysis was conducted to determine factors associated with the acceptance of a COVID-19 vaccine while adjusting for other potential predictors. Intention to receive the vaccine was used as the dependent variable (“1” for intention to receive the vaccine and “0” for unwillingness or undecidedness about being vaccinated). Sociodemographics, health-related factors, public trust and satisfaction, and HBM and TPB dimensions were included as independent variables. Only significant variables (*p* < 0.05) in univariate analysis were included in the regression model. These independent variables were divided into five blocks and entered sequentially using a series of models and a forward stepwise regression approach. Sociodemographics and health-related variables were entered into blocks 1 and 2, respectively. The trust/satisfaction with health authorities variable was entered in block 3. The HMB and TPB dimensions were entered in blocks 4 and 5, respectively. Cohen’s *d* values were used to estimate the magnitudes of each effect [41]. A two-tailed test with a significance level of *p* < 0.05 was judged statistically significant. Reasons for refusing/hesitating to take the vaccine were described as frequencies. All analyses were conducted using IBM SPSS software version 26.

## 3. Results

### 3.1. Sociodemographic Characteristics and COVID-19 Vaccine Acceptance

The sociodemographic and health-related characteristics of the participants are provided in Table 1. Most were female (61.3%), relatively young (mean age: 24.3, standard deviation (SD): 3.7), and holders of at least a bachelor’s degree (92.3%). More than half were from the Khulna division. Around 65% were urban residents and 77.4% lived with family members. Only 1.7% were healthcare professionals. Most were students (70.9%). Half (50.5%) had been diagnosed with COVID-19 at some point. The majority (68.1%) were in a normal weight range (BMI: 25.0–29.9). The majority did not have hypertension, were not overweight, and did not smoke. Nearly 50% reported a long-standing illness. Two-thirds could not remember whether they had taken seasonal influenza vaccines or not, although almost all received childhood vaccines (96.9%).

The proportion of individuals that displayed intentions to get a COVID-19 vaccination was around 85% (*n* = 460). A comparison of demographic and health-related variables by vaccine acceptance indicated that five factors showed differences between vaccine acceptance groups (Table 1). Older respondents (χ^2^ = 36.494, *p* = 0.019), students (χ^2^ = 9.969, *p* = 0.044), respondents who did not smoke (χ^2^ = 4.363, *p* = 0.037), respondents in a normal weight range (χ^2^ = 3.086, *p* = 0.041) and respondents who received childhood vaccinations (χ^2^ = 5.426, *p* = 0.020) were more likely to accept a COVID-19 vaccination.

### 3.2. Univariate Analyses of Trust/Satisfaction with Authorities and Health Behavior Variables

The findings of univariate analyses of public trust and satisfaction, HBM dimensions, and TPB dimensions with respect to COVID-19 vaccine acceptance are presented in Table 2. Significant differences were found between the two groups for public trust/satisfaction with authorities (*p* < 0.010). Further, all dimensions of the HBM except health motivation exhibited significant differences between groups. Compared to the undecided/unwillingness group, individuals who intended to get vaccinated were more susceptible to COVID-19 (χ^2^ = 57.11, *p* < 0.001), perceived a higher severity of the virus (χ^2^ = 9.97, *p* < 0.010), found greater benefits of vaccination (χ^2^ = 54.04, *p* < 0.001), and possessed higher levels of cues to action (χ^2^ = 57.03, *p* < 0.001). By contrast, participants who were undecided/unwilling to get vaccinated revealed higher levels of perceived barriers than their counterparts (χ^2^ = 14.54, *p* < 0.001). Significant differences were also found between groups for all TPB dimensions except behavior control (χ^2^ = 0.04, *p* = 0.834). People who intended to get vaccinated stated greater subjective norms than their counterparts (χ^2^ = 29.66, *p* < 0.001). Respondents who had stronger unfavorable attitudes toward the COVID-19 vaccine were also less likely to get vaccinated (χ^2^ = 10.02, *p* < 0.010). Finally, individuals with less self-efficacy regarding their ability to prevent infection displayed stronger intentions to get vaccinated (χ^2^ = 40.63, *p* < 0.001).

### 3.3. Multivariate Analysis of Factors Associated with COVID-19 Vaccine Acceptance

The results of the hierarchical logistic regression analysis are displayed in Table 3. The first model with only sociodemographic characteristics explained 4% of the variance. Students, among other professions, were significantly more likely to intend to get the COVID-19 vaccine (odds ratio (OR) = 2.01, 95% confidence interval (CI): 1.10–3.67, *d* = 0.69).

The second model that included health-related factors added 5% of explained variance. Smoking status, weight status, and childhood vaccinations were significant predictors of COVID-19 vaccine acceptance. Compared to underweight respondents, obese respondents were less willing to get vaccinated against COVID-19 (OR = 0.25, 95% CI = 0.06–0.89, *d* = 1.40). Respondents who had a habit of smoking were also less likely to take COVID-19 vaccine (OR = 0.56, 95% CI = 0.33–0.96, *d* = 0.57). On the other hand, people who received childhood vaccines exhibited a 3.3-times greater likelihood of accepting a COVID-19 vaccine than people without childhood vaccines (95% CI: 0.99–10.99, *d* = 1.17).

Model 3 added public trust and satisfaction in health and political authorities, which added 5% to the explained variance. Respondents with greater trust/satisfaction with authorities were more likely to intend to get vaccinated (OR = 1.95, 95% CI: 1.39–2.75, *d* = 0.67).

Model 4 introduced HBM dimensions, which added 23% to the explained variance. Four dimensions showed significant associations with intentions to be vaccinated (*p* < 0.010). Perceived susceptibility predicted willingness to be vaccinated (OR = 1.78, 95% CI = 1.26–2.45, *d* = 0.57), as did perceived benefits (OR = 2.00, 95% CI = 1.29–3.09, *d* = 0.69) and cues to action (OR = 2.05, 95% CI = 1.3–3.17, *d* = 0.72). On the other hand, perceived barriers expressed a negative relationship with vaccine acceptance (reverse score: OR = 0.49, 95% CI = 0.34–0.71, *p* < 0.001, *d* = −0.70).

The fifth and final model included TPB dimensions, which added 7% to the explained variance (final adj. R^2^ = 0.44). Only one factor exhibited significant associations with vaccine acceptance. Greater self-efficacy regarding ability to prevent infection without the vaccine showed a 55% reduction in willingness to get vaccinated (OR = 0.45, 95% CI = 0.33–0.64, *d* = −0.80).

### 3.4. Reasons behind Unwillingness/Indecision with Getting Vaccinated

**Figure 2:**
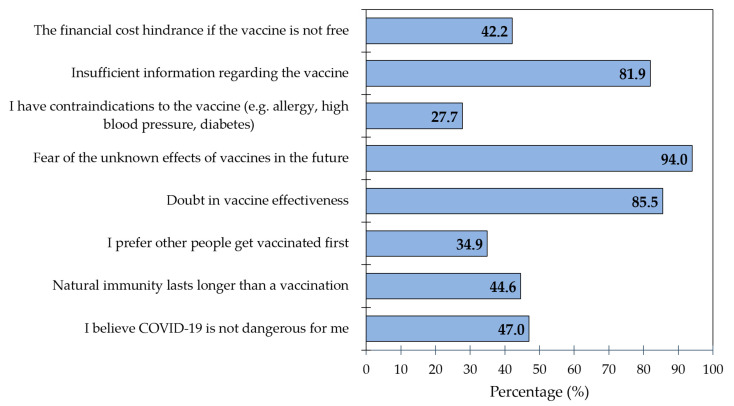
Frequency of reasons for being unwilling/undecided to get the COVID-19 vaccine (*n* = 83); COVID-19: Coronavirus disease 2019.

Overall, 15% of the respondents expressed unwillingness or uncertainty to be vaccinated against COVID-19. Most of these people (>80%) reported three reasons for vaccine hesitancy: fear of unknown future effects (94.0%), doubt in effectiveness (85.5%), and insufficient information (81.9%) (Figure 2). Nearly half of the respondents believed that COVID-19 was not dangerous for them, that natural immunity lasts longer than vaccination, and that the vaccine was not free.

## 4. Discussion

To date, authorities have fought the COVID-19 pandemic largely through emphasizing hygiene practices and social distancing. However, to establish global immunity, people must be willing to get vaccinated. Our findings demonstrated that eight out of every ten Bangladesh adults intend to be vaccinated. Hierarchical logistic regression models showed that being a student, having received a childhood vaccine(s), being satisfied with/trusting authorities, being concerned with contracting COVID-19 without a vaccine (perceived susceptibility), believing a vaccine would be beneficial, reporting fewer barriers of receiving the vaccine, reporting cues to action (i.e., doctors, families, and friends recommending the vaccine), and showing less self-efficacy (e.g., ability to avoid infection without a vaccine) predicted vaccine acceptance. Smoking and obesity negatively predicted COVID-19 vaccine acceptance. Among respondents who were hesitant/unwilling to get the vaccine, the most commonly reported reasons were fear of the unknown future effects of the vaccine, doubt in the vaccine’s effectiveness, and insufficient information regarding the vaccine.

Regarding our study’s high prevalence of COVID-19 vaccine intentions, this finding is generally consistent with other observations. Similarly, high vaccine acceptance rates have been noticed among the general population of India (89.3%), healthcare workers in Kuwait (83.3%), Australian citizens, Israeli adults, the general population of France (77.6%), pregnant women of China (77.4%), and French healthcare workers (76.9%) [9,32,42,43,44,45,46,47].

Even in Bangladesh, a former study conducted among people aged 18 years and older reported acceptance rates of 74.6% that were comparable to our study [20]. The Bangladesh government inaugurated the first COVID-19 vaccination campaign on 27 January 2021 during the peak time of viral infections and deaths [48]. Willingness to get vaccines was around 60–70% even before the vaccines were introduced, and this ratio has been relatively consistent over time [49,50,51].

The majority of the vaccine acceptance group expressed that the vaccine would defend against COVID-19 infection and would control or stop viral transmission [19,52]. According to Yan et al., the low tendency to accept vaccines has been consistent in Hong Kong across time [53]. Most of the people in the aforementioned studies were doubtful about the trust, safety, and effectiveness of the vaccine. Of note, some considered other activities were more important than being vaccinated. A study in Chile stated that only 49% of participants were willing to vaccinate, despite the vaccine being 95% effective, due to worry about side-effects and lack of proper knowledge [54]. In the case of Bangladesh, during January 2021, healthcare workers expressed comparatively low intentions to receive COVID-19 vaccines compared to the general population, despite having average to good knowledge levels about the vaccine [55]. This is a particularly worrisome issue for the public health sector, since after the official vaccination campaign, healthcare workers were the targeted group to be vaccinated first.

Our study found that age and occupation, among other sociodemographic variables, predicted differences in vaccination intentions. These findings are congruous with another rapid national study of Bangladesh carried out in the early stages of the vaccination program [56]. In that study and in ours, students were more likely to intend to get a COVID-19 vaccine than some other occupations/employment statuses. Higher intentions by students to get vaccinated were also identified by Akiful Haque et al. [57]. These findings might be explained by students being more aware of their own and their friends’ safety if they study in confined spaces. Another possible explanation is that the Bangladesh government strove to inoculate students to avoid educational disruptions and that students in turn were more willing to accept the vaccine [58].

Smoking behavior being a predictor of vaccine hesitancy is generally in line with past research as well. For instance, a recent study from the UK found that current smokers were 1.43-times more likely to be uncertain about the COVID-19 vaccine than respondents who never or formerly smoked [59]. A study in Kuwait also reported that current smokers were less likely to accept the vaccine than non-smokers [60]. Associations between smoking and vaccination have been similar for other viruses (e.g., influenza) [61,62]. Some smokers believe that smoking makes no or very little difference in the risk of developing severe COVID-19 infections [63]. We cannot underestimate our concern that if smokers considered this potential protective effect to be true, they might misinterpret it as an indication that the vaccine offers little protection [59]. In addition, smokers tend to show other health-degrading behaviors that could result in low vaccine acceptance [61,62]. Thus, policymakers and governments should increase public awareness of the benefits of vaccination among smokers and dispel misconceptions about smoking providing protection against COVID-19 [64].

People who were obese were less likely to accept the COVID-19 vaccination. Our findings are in accordance with a study from Canada showing that obese people were more ambivalent about COVID-19 vaccination but contrast with a study from Kuwait that found overweight and obese people were more likely to get the vaccine [60,65]. Such variations could be explained by differences in public perceptions and health beliefs about vaccination between countries. There is growing evidence that obesity is a risk factor for death by COVID-19 and that the vaccine may not work well for obese people [66]. The first few months of vaccine trials have found some evidence that people with abnormal BMI levels showed short-term vaccination efficacy [67]. Therefore, these findings may provide some rationale for why obese respondents showed vaccine hesitancy.

Higher rates of COVID-19 vaccine acceptance were reported among respondents who received a childhood vaccination(s). Possible reasons behind this relationship include positive inoculation experiences, positive perceptions of vaccination services, and perceived social responsibility [57]. In our study, vaccine acceptance in Model 3 (absent of Health Behavior Model variables) revealed that trust/satisfaction with authorities’ ability to control COVID-19 transmission showed a two-fold increase in vaccine acceptance. This finding is congruent with previous research in Hong Kong, which found a three-fold increase in intentions to vaccinate [53]. In contrast to the Hong Kong study, we found public trust/satisfaction was no longer associated with vaccine acceptance in fully adjusted models. Further research is needed to differentiate potential confounding and mediating associations between trust/satisfaction with authorities, health behavior models, and vaccine acceptance.

Satisfaction with health authorities was found to be a significant predictor of COVID-19 vaccine acceptance, which also aligns with past research [68]. Another robust study across 12 countries found that respondents in Germany and the UK were more likely to get vaccinated if they trusted political leaders. Moreover, respondents across all countries reported greater vaccine acceptance when they trusted in national scientific and medical advisors [69]. Further research is necessary to decouple the unique effects of satisfaction with health authorities and health beliefs, attitudes, and perceptions in explaining vaccine acceptance.

Regarding our findings with respect to the dimensions of the HBM, vaccine acceptance may be related to respondents’ perceived susceptibility to COVID-19, perceived benefits of the vaccine, fewer perceived barriers of receiving the vaccine, and cues to action from doctors, family, and friends. These observations are supported by other studies’ findings about influenza vaccination acceptance in Bangladesh and elsewhere [43,53,68,70]. For instance, in a study of the capital city of Bangladesh, residents were likely to vaccinate themselves and others if they were concerned about being infected by the virus [21]. Of note, perceived severity of COVID-19 infection was not associated with vaccine acceptance in adjusted models. Previous research also found mixed results regarding the potential influence of how severe a respondent believes a COVID-19 infection would be and vaccine acceptance [35,71,72]. Collectively, it appears that the perceived likelihood of preventing infection is a more consistent predictor of vaccine acceptance than perceived likelihood of a severe infection.

Past research also supports our findings regarding cues to action. Recommendations from doctors, politicians, governmental health authorities, and family/friends can be a driving force for vaccine acceptance [69,73,74]. For example, one Malaysian study reported that participants wanted to vaccinate only when other closely related people were vaccinated [34]. Our findings regarding fewer perceived barriers predicting vaccine acceptance represents a second driving force for vaccination [75,76] and are supported by research in Hong Kong, where perceived barriers related to vaccine access were negatively associated with vaccine acceptance [35]. Furthermore, the Hong Kong study reported that perceived harm negatively predicted vaccine intentions. Overall, dimensions of the HBM appear to be a useful for understanding vaccine uptake and behavioral intentions [77].

The final model of the study considered TBP variables and found that self-efficacy predicted COVID-19 vaccine acceptance. A recent study on Israel somewhat supports our findings, although the authors found that both subjective norms and self-efficacy explained vaccine intentions [32]. Elsewhere, attitudes and subjective norms have been shown to predict vaccine intentions [40,78,79]. This discrepancy in results may relate to Bangladeshi peoples’ expectations regarding who should be vaccinated first. For the first phase of the vaccine rollout, the Bangladesh government issued a priority list that included frontline workers and persons over the age of 40 [20]. The current study included people aged 18 or older and mostly students, who were somewhat lower on the vaccination priority list than other groups (i.e., healthcare workers). Interestingly, the effects of trust/satisfaction with authorities were attenuated to null after introducing HBM and TPB dimensions. In this case, worry and concern regarding COVID-19 may be a factor that causes people’s beliefs to change [80].

Amongst respondents who expressed vaccine hesitancy, we found that doubt in vaccine effectiveness and inadequate information regarding the vaccine were the most commonly stated reasons respondents gave for not accepting the vaccine. Similar reasons have been observed in other studies of Bangladeshi citizens [49,55,81]. The concern about side effects could have been accelerated and exaggerated by hearing stories directly or second-hand from recently vaccinated people. Parvej et al. observed Bangladeshi citizens reported numerous common side effects of the vaccine, including fever, muscle pain, pain in the injection site, headache, and malaise [52]. Rumors and misleading information about vaccine side effects have also circulated in traditional and social media, in part by anti-vaccine groups who propelled conspiracy theories regarding gene alteration, microchips, and haram ingredients (e.g., pork fat for Muslim) [23,24,82]. Taken together, these findings suggest that providing sufficient and accurate information to the general people about the COVID-19 vaccine, as well as substantial evidence of the vaccine’s safety and effectiveness, may be important in reducing vaccine hesitancy.

### 4.1. Implications

The key step to slowing down the spread of infection and restoring the economy is to vaccinate the masses. The Bangladesh Government has already done some commendable work towards vaccinating its citizens, but these efforts have not yet been sufficient to achieve population immunity. Successful vaccination can be partly achieved through an uninterrupted supply of vaccines and appropriate vaccination infrastructure. However, another main challenge is persuading people to get vaccinated. Our study identified some important beliefs and behavioral factors related to vaccine acceptancy. These generally align with the WHO’s Technical Advisory Group on Behavioral Insights and Sciences for Health recommendation on social and behavioral drivers of COVID-19 vaccination (https://apps.who.int/iris/handle/10665/337335, accessed on 14 November 2021).

Thus, our study could pave the way for health authorities in Bangladesh to design appropriate interventions for increasing vaccine acceptance. For example, influential persons, such as healthcare staff and family members, should give testimonials on the benefits of getting the vaccine and distribute their experiences over social media and other platforms to promote social norms. Public leaders and celebrities who have already been vaccinated can also share their experiences widely in the media. Our study findings could also help policymakers identify priority groups that need special attention in vaccination campaigns. To increase awareness of the severity of COVID-19 infection, testimonials of affected family members could be shared. Along with COVID-19 prevention, the axillary benefits of immunization should be promoted. While it is difficult to control misinformation, it remains important to identify baseless rumors and myths and to disseminate accurate scientific facts about the vaccine. Additional educational campaigns could be a good option to make the acceptance process more visible.

### 4.2. Strengths and Limitations

The main strength of this study is its novelty—simultaneously applying the Health Behavior Model and Theory of Planned Behavior to understand COVID-19 vaccine acceptance. Another strength is the investigation of COVID-19 vaccine acceptance in a low/middle-income country with limited resources to otherwise control the spread of the virus. Accordingly, the current study included respondents from all eight divisions of the country studied to achieve a more representative sample of respondents and nationwide perspectives on vaccine hesitancy. However, some limitations were unavoidable. Our study design was cross-sectional, so we could not draw causal links between variables of interest. Longitudinal studies that follow people’s vaccination intentions over time may assist researchers in determining how these variables are influenced by changes in health policy and media coverage. We also carried out this survey on an online platform to avoid face-to-face interviews during the pandemic. This approach could have resulted in self-selection and response biases. An additional limitation comes from the fact that the study only reached people with the internet and connected devices (e.g., smartphone, laptop). Our sample was unable to include some uneducated and extremely poor people, among whom rates of vaccine acceptance might be different from those in our sample [83].

Further, our study findings may not generalize to some populations, such as healthcare professionals, pregnant women, and adult people with chronic diseases who can be at high risk of severe COVID-19 infection. This study also did not achieve an equal distribution of urban and rural participants, since internet access is greater in urban areas. Our study did not investigate the depth of knowledge of vaccine acceptance and some other potentially influential factors (e.g., changes in case counts) to minimize the respondent burden and excessive questionnaire length. These variables should be addressed in future research when possible. Finally, the findings of this study were based on self-reported information, which was subject to information bias. Ultimately, future studies should focus on more stringent sampling methods to achieve nationwide representative samples and more balanced socio-demographic groups to determine vaccine acceptance and its factors.

## 5. Conclusions

A successful vaccination program relies on high rates of vaccine acceptance to achieve herd immunity. Our study found that 85% of Bangladeshi citizens intended to receive the vaccine. In fully adjusted models, students and respondents with more normal body weights were more likely to report vaccine acceptance. Psychological traits also emerged as predictors of vaccine acceptance, including perceived susceptibility to infection, benefits of vaccination, fewer barriers to vaccination (i.e., side-effects), recommendations from health authorities/friends/family, and low self-efficacy in preventing infection. Based on these results, it is recommended that governmental and health authorities raise public confidence in COVID-19 vaccinations considering the influence of these beliefs on vaccine hesitancy.

## Figures and Tables

**Table 1:** Descriptive statistics of study respondents by intention to get the COVID-19 vaccination (*N* = 543).

Variables	Total *N* = 543 (%)	COVID-19 Vaccine Acceptance		χ^2^	*p* Value
		Intended (*n* = 460) (%)	Undecided/Unwilling (*n* = 83) (%)		
**Sociodemographic characteristics**					
*Gender*				0.264	0.607
Male	210 (38.67)	180 (39.13)	30 (36.14)		
Female	333 (61.33)	280 (60.87)	53 (63.86)		
Age	24.29 (±3.67)	24.14 (±2.92)	25.13 (±6.35)	36.494	0.019
*Education*				2.670	0.263
Less than college education	42 (7.73)	32 (6.96)	10 (12.05)		
Bachelor’s degree	272 (50.09)	231 (50.21)	41 (49.40)		
Beyond Bachelor’s degree	229 (42.17)	197 (42.83)	32 (38.55)		
*Administrative division*				4.680	0.699
Barishal	26 (4.79)	24 (5.22)	2 (2.41)		
Chittagong	35 (6.45)	29 (6.30)	6 (7.23)		
Dhaka	134 (24.68)	111 (24.13)	23 (27.71)		
Khulna	285 (52.49)	243 (52.83)	42 (50.60)		
Mymensingh	8 (1.47)	8 (1.74)	0 (0.00)		
Rajshahi	27 (4.97)	21 (4.57)	6 (7.23)		
Rangpur	25 (4.60)	21 (4.57)	4 (4.82)		
Sylhet	3 (0.55)	3 (0.65)	0 (0.00)		
*Place of residence*				0.547	0.460
Urban	353 (65.01)	302 (65.65)	51 (61.45)		
Rural	190 (34.99)	158 (34.35)	32 (38.55)		
*Living status*				2.955	0.228
With family members	420 (77.35)	360 (78.26)	60 (72.29)		
With non-family members	84 (15.47)	66 (14.35)	18 (21.69)		
Alone	39 (7.18)	34 (7.39)	5 (6.02)		
*Occupation*				9.969	0.044
Unemployed	84 (15.46)	64 (13.91)	20 (24.10)		
Student	375 (69.06)	328 (71.30)	47 (56.62)		
Public sector	7 (1.29)	7 (1.52)	0 (0.00)		
Private sector	39 (1.18)	31 (6.74)	8 (9.64)		
Self-employed	38 (7.00)	30 (6.52)	8 (9.64)		
*Healthcare worker*				1.697	0.428
No	499 (91.90)	421 (91.52)	78 (93.98)		
Healthcare student	35 (6.45)	30 (6.52)	5 (6.02)		
Healthcare professional	9 (1.66)	9 (1.96)	0 (0.00)		
**Health related characteristics**					
*Tested positive for COVID-19*				0.553	0.457
No	269 (49.54)	231 (50.52)	38 (45.78)		
Yes	274 (50.46)	229 (49.78)	45 (54.22)		
*BMI*				8.244	0.041
Underweight	49 (9.02)	45 (9.78)	4 (4.82)		
Normal weight	370 (68.14)	310 (67.39)	60 (72.29)		
Overweight	88 (16.21)	79 (17.17)	9 (10.84)		
Obesity	36 (6.63)	26 (5.65)	10 (12.05)		
*Long-standing illness(es)*				0.006	0.938
No	290 (53.41)	246 (53.48)	44 (53.01)		
Yes	253 (46.59)	214 (46.52)	39 (46.99)		
*Hypertension*				2.484	0.115
No	516 (95.03)	440 (96.65)	76 (91.57)		
Yes	27 (4.97)	20 (4.35)	7 (8.43)		
*Overweight*				0.048	0.826
No	487 (89.69)	412 (89.57)	75 (90.36)		
Yes	56 (10.31)	48 (10.43)	8 (9.64)		
*Smoking*				4.363	0.037
No	415 (76.42)	359 (78.04)	56 (67.46)		
Yes	128 (23.58)	101 (21.96)	27 (32.54)		
*Childhood vaccination(s)*				5.426	0.020
No	17 (3.13)	11 (2.39)	6 (7.23)		
Yes	526 (96.87)	449 (97.61)	77 (92.77)		
*Seasonal influenza vaccination*				1.355	0.852
Never	140 (25.78)	122 (26.52)	18 (21.69)		
Last year	5 (0.92)	4 (0.87)	1 (1.20)		
Current flu season	2 (0.37)	2 (0.43)	0 (0.00)		
Annually	11 (2.03)	9 (1.6)	2 (2.41)		
Can’t remember	385 (70.90)	323 (70.22)	62 (74.70)		

COVID-19: Coronavirus disease 2019; BMI: Body mass index; *p* values were calculated using the Kruskal–Wallis test/chi-square test.

**Table 2:** Univariate analysis of trust/satisfaction with health authorities as well as Health Behavior Model (HBM) and Theory of Planned Behavior (TPB) dimensions regarding willingness to receive the COVID-19 vaccine.

Variables	Total (*N* = 543)	COVID-19 Vaccine Acceptance		χ^2 a^	*p* Value
		Intended (*n* = 460)	Undecided/Unwilling (*n* = 283)		
	Mean (SD)	Mean (SD)	Mean (SD)		
Trust/satisfaction with authorities	1.01 (0.83)	1.07 (0.84)	0.66 (0.67)	14.56	0.000 ***
*HBM*					
Perceived susceptibility	3.44 (1.01)	3.58 (0.97)	2.69 (0.87)	57.11	0.000 ***
Perceived severity	3.60 (0.87)	3.64 (0.89)	3.39 (0.73)	9.97	0.002 **
Perceived benefits	3.45 (0.87)	3.56 (0.83)	2.83 (0.84)	54.04	0.000 ***
Perceived barriers (reverse coded)	3.40 (0.85)	3.35 (0.83)	2.72 (0.89)	14.54	0.000 ***
Cues to action	3.19 (0.84)	3.30 (0.83)	2.59 (0.68)	57.03	0.000 ***
Health motivation	3.28 (0.91)	3.28 (0.91)	3.30 (0.95)	0.14	0.712
*TPB*					
Attitude	2.52 (1.05)	2.48 (1.07)	2.80 (0.91)	10.02	0.002 **
Subjective norms	3.57 (0.89)	3.64 (0.89)	3.16 (0.76)	29.66	0.000 ***
Perceived behavioral control	3.89 (0.96)	3.89 (0.95)	3.92 (0.99)	0.04	0.834
Self-efficacy (reverse coded)	2.50 (1.01)	2.39 (1.01)	3.10 (0.79)	40.63	0.000 ***

Data are presented as mean (±SD). ^a^ Kruskal–Wallis test; ** *p* < 0.010, *** *p* < 0.001.

**Table 3:** Hierarchical logistic regression analysis of COVID-19 vaccine acceptance in Bangladesh (*N* = 543).

Predictors	Odds Ratio (95% Confidence Interval), Effect Size ^§^				
	Model 1	Model 2	Model 3	Model 4	Model 5
**Sociodemographic factors**					
Age	0.96 (0.90–1.03), −0.42	0.96 (0.89–1.01), −0.04	0.95 (0.89–1.02), −0.04	0.99 (0.92–1.07), −0.01	0.99 (0.91–1.07), −0.01
*Occupation*					
Unemployed	Ref.	Ref.	Ref.	Ref.	Ref.
Student	2.01 * (1.10–3.67), 0.69	2.06 * (1.11–3.83), 0.72	1.89 * (1.00–3.55), 0.63	2.41 * (1.12–5.18), 0.88	2.56 * (1.16–5.67), 0.94
Public sector	3.34 (0.00–6.98), 0.78	4.2 (0.00–4.65), 0.83	4.8 (0.20–5.67), 0.79	5.67 (0.00–9.78), 0.75	4.23 (0.01–10.09), 0.82
Private sector	1.28 (0.50–3.24), 0.25	1.40 (0.54–3.65), 0.34	1.38 (0.52–3.65), 0.32	0.86 (0.28–2.59), −0.15	0.99 (0.31–3.16), −0.01
Self-employed	1.41 (0.53–3.79), 0.35	1.71 (0.63–4.64), 0.54	1.62 (0.59–4.51), 0.48	2.25 (0.65-7.79), 0.81	3.02 (0.82–11.20), 1.10
**Health related factors**					
*BMI*					
Underweight		Ref.	Ref.	Ref.	Ref.
Normal weight		0.54 (0.18–1.59), −0.61	0.56 (0.18–1.69), −0.57	0.40 (0.12–1.36), −0.91	0.41 (0.12–1.45), −0.89
Overweight		0.97 (0.27–3.42), −0.03	0.97 (0.27–3.49), −0.27	0.64 (0.15–2.67), −0.45	0.60 (0.14–2.71), −0.50
Obesity		0.25 * (0.06–0.89), −1.40	0.25* (0.06–0.91), −1.39	0.17* (0.04–0.76), −1.78	0.14 * (0.02–0.68), −1.98
*Smoking*					
No		Ref.	Ref.	Ref.	Ref.
Yes		0.56 * (0.33–0.96), −0.57	0.58 * (0.34–0.99), −0.54	0.66 (0.35–1.24), −0.41	0.73 (0.37–1.41), −0.31
*Childhood vaccination*					
No		Ref.	Ref.	Ref.	Ref.
Yes		3.24 * (1.12–9.32), 1.17	3.72 * (1.22–11.35), 1.31	2.62 (0.70–9.74), 0.96	2.18 (0.53–8.83), 0.78
Satisfaction with authorities			1.95 *** (1.39–2.75), 0.67	1.32 (0.89–1.96), 0.28	1.51 (0.98–2.29), 0.41
*HBM Dimensions*					
Perceived susceptibility				1.78 ** (1.26–2.45), 0.57	1.73 ** (1.20–2.51), 0.55
Perceived severity				0.68 (0.44–1.06), −0.38	0.67 (0.42–1.06), −0.40
Perceived benefits				2.00 ** (1.29–3.09), 0.69	2.02 ** (1.26–3.25), 0.71
Perceived barriers				0.49 *** (0.34–0.71), −0.70	0.63 * (0.42–0.93), −0.46
Cues to action				2.05 ** (1.3–3.17), 0.72	1.98 ** (1.21–3.26), 0.68
*TPB Dimensions*					
Attitude					0.89 (0.67–1.21), −0.11
Subjective norms					1.21 (0.78–1.88), 0.19
Self-efficacy					0.45 *** (0.33–0.64), −0.80
**Model Fit Statistics**					
Cox and Snell pseudo R^2^	0.02	0.05	0.08	0.21	0.25
Nagelkerke pseudo R^2^	0.04	0.09	0.14	0.37	0.44

Only significant variables (*p* < 0.050) in the univariate analysis were considered for the hierarchical logistic regression analysis, significant coefficients shown in bold; * *p* < 0.050, ** *p* < 0.010, *** *p* < 0.001; COVID-19: Coronavirus disease 2019; HBM: Health Belief Model; TPB: Theory of Planned Behavior; ^§^ Small effect if Cohen’s |*d*| ≤ 0.20; moderate effect if Cohen’s *d* 0.20 < |*d*| ≤ 0.50; large effect if Cohen’s |*d*| > 0.50.

## Data Availability

Data generated in this study is available by contacting the first author, Muhammad Mainuddin Patwary, if reasonably requested.
